# Determinants and Prediction Equations of Six-Minute Walk Test Distance Immediately After Cardiac Surgery

**DOI:** 10.3389/fcvm.2021.685673

**Published:** 2021-08-19

**Authors:** Basuni Radi, Ade Meidian Ambari, Bambang Dwiputra, Ryan Enast Intan, Kevin Triangto, Anwar Santoso, Budhi Setianto

**Affiliations:** ^1^Department of Cardiovascular Prevention and Rehabilitation, National Cardiovascular Center Harapan Kita, Jakarta, Indonesia; ^2^Department of Cardiology and Vascular Medicine, Faculty of Medicine, Universitas Indonesia, Jakarta, Indonesia; ^3^Adhyaksa Hospital, Jakarta, Indonesia

**Keywords:** 6-minute walk test, CABG, valve surgery, cardiac surgery, cardiac rehabilitation

## Abstract

**Background:** To date, there is no reference for a 6-min walk test distance (6-MWD) immediately after cardiac surgery. Therefore, this study aimed to identify the determinants and to generate equations for prediction reference for 6-MWD in patients immediately after cardiac surgery.

**Methods:** This is a cross-sectional study of the 6-min walk test (6-MWT) prior to participation in the cardiac rehabilitation (CR) program of patients after coronary artery bypass surgery (CABG) or valve surgery. The 6-MWT were carried out in a gymnasium prior to the CR program immediately after the cardiac surgery. Available demographic and clinical data of patients were analyzed to identify the clinical determinants of 6-MWD.

**Results:** This study obtained and analyzed the data of 1,509 patients after CABG and 632 patients after valve surgery. The 6-MWD of all patients was 321.5 ± 73.2 m (60–577). The distance was longer in the valve surgery group than that of patients in the CABG group (327.75 ± 70.5 vs. 313.59 ± 75.8 m, *p* < 0.001). The determinants which significantly influence the 6-MWD in the CABG group were age, gender, diabetes, atrial fibrillation, and body height, whereas in the valve surgery group these were age, gender, and atrial fibrillation. The multivariable regression models generated two formulas using the identified clinical determinants for patients after CABG: 6-MWD (meter) = 212.57 + 30.47 (if male gender) − 1.62 (age in year) + 1.09 (body height in cm) − 12.68 (if with diabetes) − 28.36 (if with atrial fibrillation), and for patients after valve surgery with the formula: 6-MWD (meter) = 371.05 + 37.98 (if male gender) − 1.36 (age in years) − 10.61 (if atrial with fibrillation).

**Conclusion:** This study identified several determinants for the 6-MWD and successively generated two reference equations for predicting 6-MWD in patients after CABG and valve surgery.

## Introduction

The 6-min walk test (6-MWT) is a simple, safe, and practical sub-maximum exercise test for patients with respiratory or heart diseases (1). It becomes an ordinary exercise test to measure the functional capacity and to evaluate the treatment or intervention. The 6-min walk test distance (6-MWD) can also be used as a predictor of hospitalization and death from cardiac or pulmonary diseases. This test is objective, independent, able to predict peak oxygen uptake, and well-tolerated by most patients (2–4). However, to the author's knowledge there is still no reference for 6-MWD which could be used to compare the 6-MWD in patients immediately after cardiac surgery.

In the case of post-cardiac surgeries, performing maximal stress testing is still contraindicated clinically. The first reason is the pain in the sternal region, which would then significantly cause arm-swing reduction and thus reduce the whole acceleration momentum during running uphill in the treadmill test. Another identified reason is that purposefully accelerating the heart rate after a surgery could potentially lead to safety issues, in which the heart is still undergoing a recovery phase. Therefore, submaximal stress testing through 6-MWT is generally acceptable and is recommended to be done prior to discharge in post-cardiac surgery patients. Additionally, the 6-MWT could also serve as a functional capacity testing if the patient could walk a distance of approximately 300 m, which serves an equivocal of 3.5 METs, reflecting the ability to walk and perform home activities with moderate effort. METs could be defined as the ratio of caloric consumption in an active person, compared to their basal metabolic rate at rest. METs are measured in kcal/kg/hour.

Henceforth, this study is aimed to both identify determinants of 6-MWD in patients after cardiac surgery and generate equations for 6-MWD of patients immediately after coronary artery bypass graft (CABG) and after valve surgery. The secondary objectives include exhibiting the baseline comparison between the CABG and valve surgery groups, owing to the fact that patient characteristics would differ in these two groups. It is then expected that these equations could be used to predict the level of functional capacity of patients immediately after cardiac surgery.

## Materials and Methods

This was a cross-sectional study which evaluated first 6-MWT prior to patients' participation in the cardiac rehabilitation (CR) program immediately after CABG surgery or valve surgery. Therefore, immediate 6-MWD is defined as the 6-MWT performed at initial visit to the CR program, in which the patients were recommended to attend CR within the first week after inpatient discharge. This was done in order to achieve the study aims through comparison of means and multivariable regression analysis.

### Patients

The population of this study was a cohort of patients who registered to participate in a CR program held in the Cardiovascular Prevention and Rehabilitation Unit of the National Cardiovascular Center Harapan Kita Jakarta, within January 2014 and December 2016 immediately after CABG or cardiac valve surgeries. As for external validation of the generated equations, we used a population group of patients immediately after CABG and valve surgery who registered in the CR program in the year 2018.

All patients had given complete verbal and written information and gave written consent to participate in the CR program, which also includes exercise stress testing. Ethical approval for the CR patient registry was provided from the Committee on Institutional Review Board of the National Cardiac Center “Harapan Kita” Hospital with the following registration number: LB.02.01/VII/536/KEP 027/2021. The CR program and the 6-MWT in this research is a standard procedure in the institution for all patients after cardiac surgery. Individual data of patients were retrieved from the available medical records for analysis.

Patients will be excluded when they have limitation of motion that prevents them from performing physical activities such as walking for 6 min and also pain due to multiple causes. Moreover, since this is an analysis of secondary data, incomplete values from the patient database will also be excluded from the study.

All of these open heart surgeries used thoracotomy approach and cardiopulmonary bypass (CPB) machine with cardioplegia protection technique. The CABG technique includes grafting the left internal mammary artery to the left anterior descending artery or its main branches and grafting the saphenous vein from the aorta to the other coronary branches. Meanwhile, valvular surgery technique includes valve repair or valve replacement using a mechanical valve or a bio-prosthesis valve.

The prevalence of atrial fibrillations (AFs) in the samples was obtained through electrocardiography (ECG) that has been performed during either their pre-exercise or pre-operative setting. Any new AFs identified would warrant further examination before admission into the CR and thus were not included in this registry.

### 6-Minute Walk Test

After being registered, the patients attended a pre-participation orientation for the CR program, medical assessment, and educational session and performed the 6-MWT when no contraindication was observed. Extracardiac conditions that hinder active walking, such as acute pain, and neuromusculoskeletal disabilities are classified as relative contraindications to 6-MWT.

The 6-MWT were carried out once before the participation in the CR program in the gymnasium with a standardized protocol at a 30-m gymnasium corridor (1).

The patients were instructed to walk as far as possible within 6 min. Standard verbal encouragement was gently provided. The remaining time was announced every minute, and every 15 s within the last minute. The patients could stop walking if any symptoms appeared, such as severe dyspnea, dizziness, fatigue, angina, or severe skeletal muscle pain; afterwards they could resume walking as soon as possible until the 6-min test time is attained. Additionally, the test will also be halted when the Borg rating of the perceived exertion scale is 15 out of 20, allowing the patient to rest and thus ensuring that these tests are performed on submaximal state. Their heart rate and rhythm were monitored using ECG telemonitor, while distance was recorded in meters.

Most patients attend the CR 1 week after discharge, and due to these relatively fast timings of CR initiation, the patients were instructed to perform 6-MWT only once. This was done in order to avoid over-exhaustion in these patients, as most patients tend to not resume daily activities before attending the CR program. Although some patients had performed 6-MWT pre-discharge when possible, these data were not included in this study, as these were done only to allow clinicians to prescribe better lifestyle modifications prior to CR participation and achieve better functional goals.

### Statistical Analysis

The demographic data, atherosclerotic risk factors, body height, body weight, left ventricle ejection fraction (LVEF), heart rhythm, length of stay during hospitalization, current medications, 6-MWD, and other relevant data of the CR program were retrieved for the analysis. The distribution of numeric data was evaluated with the Kolmogorov–Smirnov test and expressed with mean ± standard deviation when normally distributed and median (minimum–maximum) when the data are non-parametric.

The available data of 6-MWD of all the patients were used to identify the determinants of 6-MWD and to generate equations of 6-MWD reference either in the CABG group or in the valve surgery group.

Variables with *p*-value < 0.1 in univariable analysis that were seen correlated with 6-MWD (ejection fraction, body height, and body weight) were included in the multivariable regression model to analyze the magnitude of influence of the variables to the 6-MWD.

All multivariable regression analysis assumptions were checked before inclusion into the final model. For the assumption, linear relationship between independent and dependent variables was tested by Pearson correlation and linear regression test, multicollinearity by correlation coefficient below 0.7, autocorrelation by calculated Durbin Watson test, standardized residual normal distribution by Kolmogorov–Smirnov test, and homoscedasticity of variance by Levene's test of equality of error variances.

The equations were generated from the model using determinants which significantly related with 6-MWD. For the comparison with healthy persons, we used a predictive equation for reference value of 6-MWD which was generated from a study by Zou et al. (5), who used a similar procedure with this study and from an Asian ethnic population.

For internal validation, we used the original population (year 2014–2016). Meanwhile, for external validation, we used a similar population group of patients after cardiac surgery who registered in the CR program in the year 2018 which consisted of 412 patients after CABG and 324 patients after valve surgery.

The mean difference was tested using Student's *t*-test for normally distributed variables and Mann–Whitney test if proven otherwise. Pearson's product moment correlation coefficient was applied to test the correlation between predicted and observed 6-MWD in the original population and in the other population from year 2018. A Bland–Altman plot was also used to describe the variance of difference and the limit agreement of difference between predicted and observed 6-MWD by plotting the mean difference against the observed 6-MWD.

## Results

### Clinical Characteristics of the Patients

A total of 2,302 patients registered consecutively to participate in the exercise-based CR program; among those, the data of 161 patients were incomplete, accruing for 127 CABG patients and 32 valve surgery patients being excluded from the study. Therefore, this study consisted of 2,141 patients with complete data who underwent 6-MWT after CABG surgery (*n* = 1,509) or after valve surgery (*n* = 632) in January 2014 to December 2016. The patients were hospitalized for 9 ± 5 days for their surgical procedures. Most of the patients were male (75%), with normal systolic function (median left ventricle ejection function 58%) and sinus rhythm (86.1%) on their ECG.

The 6-MWTs were carried out prior to the exercise program without adverse event. The baseline characteristics (Table 1) revealed a comparable length of stay between groups. However, the patients in the CABG group were older and male dominant, had lower ejection fraction and lower prevalence of AF, but had a higher prevalence of co-morbidities regarding atherosclerotic risks as compared to the patients in the valve surgery group.

**Table 1:** Baseline characteristic of patients after cardiac surgery.

**Variables**	**Total (*n* = 2,141)**	**CABG surgery (*n* = 1,509)**	**Valve surgery (*n* = 632)**	***P***
Age, years (median)	53 (16–80)	58 (20–80)	43 (16–71)	<0.001^a^
Male gender, *n* (%)	1,613 (75)	1,291 (86)	323 (51)	<0.001^c^
Systolic BP (median)	109 (72–197)	112 (72–197)	106 (73–172)	<0.001^a^
Diastolic BP (median)	65 (32–99)	66 (32–99)	65 (40–99)	0.121^a^
Body height, cm (median)	163 (135–185)	164 (138–185)	161 (135–183)	<0.001^a^
Body weight, kg (median)	58 (29–120)	66 (30–115)	53 (29–120)	0.001^a^
Diabetes, *n* (%)	702 (32.7)	665 (44)	37 (5.8)	<0.001^c^
Dyslipidemia, *n* (%)	846 (39.5)	813 (54)	33 (6.7)	<0.001^c^
Smoker, *n* (%)	903 (42.2)	804 (53)	99 (18.9)	<0.001^c^
LVEF, % (median)	58 (12–93)	56 (12–81)	60 (18–93)	<0.001^a^
ECG with AF, *n* (%)	298 (13.9)	34 (2.3)	264 (41.7)	<0.001^c^
Length of stay, day (mean)	9 ± 4.9	9 ± 5	9 ± 4.8	0.488^b^
6-MWD in meters (mean)	321.5 ± 73.2	313.6 ± 75.8	327.8 ± 70.5	0.001^b^

*Values are mean ± SD, median (minimum–maximum) or frequency (%). CABG, coronary artery bypass graft; BP, blood pressure; LVEF, left ventricle ejection fraction; ECG, electrocardiography; AF, atrial fibrillation; 6-MWD, 6-min walk test distance; p, for comparison between the CABG surgery group and the valve surgery group*.

a *Mann–Whitney U-test*.

b *Independent t-test*.

c *Chi square test*.

### 6-Minute Walk Test Performance

**Figure 1:**
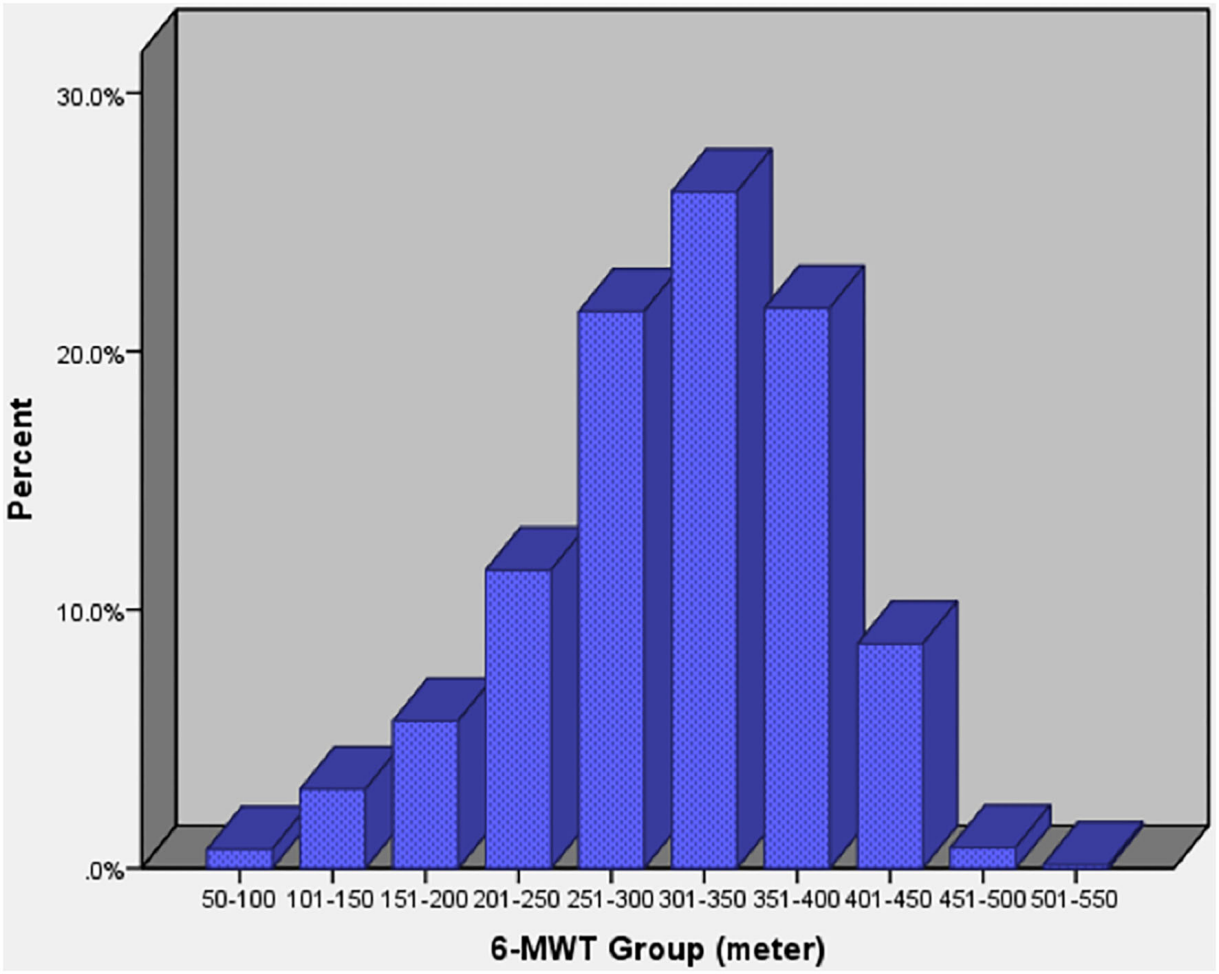
Distribution of 6-MWD of patients after cardiac surgery. 6-MWD, 6-min walk test distance.

The mean 6-MWD of all patients was 321.5 ± 73.2 m, which ranged from 60 to 488 m as seen in Figure 1. It was 57.4 ± 12.8% (range: 13–91%) of the predicted reference distance of the healthy population (5). It could be evidently seen that post CABG subjects had lower 6-MWDs as compared to those after valve surgery (313.6 ± 75.8 vs. 327.8 ± 70.5, *p* = 0.001).

In multivariable regression analysis, we entered the variables which were assumed to have influence on functional capacity, and variables from univariable analysis which significantly correlated with the 6-MWD with *p* < 0.1 (Supplementary Table 1). This analysis revealed that gender, age, diabetes status, body height, and heart rhythm were significantly related with 6-MWD in the patients after CABG; meanwhile, for patients after valve surgery, slightly variable differences were observed in gender, age, and heart rhythm.

Predictive equations of 6-MWD in both patient groups were generated from the determinants (Table 2) and were seen to be significantly related with 6-MWT (*p* < 0.05).

**Table 2:** Multivariable linear regression model of 6-MWD post cardiac surgery.

	**CABG surgery**			**Valve surgery**		
**Model**	**Unstandardized coefficients**		***P***	**Unstandardized coefficients**		***P***
	**B**	**Standard error**		**B**	**Standard error**	
(Constant)	212.57	49.55	<0.001	371.05	9.99	<0.001
Male gender	30.47	5.19	<0.001	37.98	5.57	<0.001
Age	−1.62	0.23	<0.001	−1.36	0.22	<0.001
Diabetes	−12.68	3.59	<0.001	-	-	-
Body height	1.09	0.29	<0.001	-	-	-
AF	−28.36	11.05	0.027	−10.61	5.60	0.049
Adjusted R^2^	15.3%			14.6%		
ANOVA *p*-value	<0.001			<0.001		
Durbin Watson	2.040			2.037		
Standardized residual Kolmogorov–Smirnov	0.342			0.479		
Levene's test equality of error variances	0.751			0.848		

*This multivariable regression model explained 15.3 and 14.6% of 6-MWD after CABG and valve surgery, respectively. Homogeneity of residual and homoscedasticity assumption by Kolmogorov-Smirnov test and Levene's test are fulfilled (p > 0.05)*.

*6-MWD, 6-min walk test distance, CABG, coronary artery bypass graft; gender, male = 1, female = 0; age, year; diabetes, no = 0; body height, cm; AF, atrial fibrillation = 1; sinus rhythm = 0*.

For the patients after CABG, the equation is as follows:

$$\begin{array}{l} 6-\text{MWD}\left(\text{in meters}\right)=212.57-1.62\left(\text{age in year}\right)\\ +1.09\left(\text{body height in cm}\right)+30.47\left(\text{if male gender}\right)\\ -12.68\left(\text{if with diabetes}\right)-28.36\left(\text{if with atrial fibrillation}\right) \end{array}$$

And for the patients after valve surgery, the equation is as follows:

$$\begin{array}{l} 6-\text{MWD}\left(\text{in meters}\right)=371.05-1.36\left(\text{age in year}\right)\\ +37.98\left(\text{if male gender}\right)-10.61\left(\text{if with atrial fibrillation}\right) \end{array}$$

### Internal and External Validation

We applied the equation to calculate predicted 6-MWD and compared the result with the observed 6-MWD in the original population (2014–2016) and in the population from 2018 for internal and external validation. The comparison seemed to show no significant difference between predicted and observed distances (Table 3).

**Table 3:** Comparison between predicted and observed 6-MWD for internal and external validation.

	**Population (year)**	**Mean observed 6-MWD**	**Mean predicted 6-MWD**	***P*-value**
CABG	2014–2016	313.59	313.23	0.87
	2018	337.28	331.89	0.11
Valve surgery	2014–2016	327.75	327.91	0.93
	2018	328.13	326.70	0.59

*p-value of difference between observed and predicted 6-MWT distance. 6-MWD, 6-min walk test distance*.

**Figure 2:**
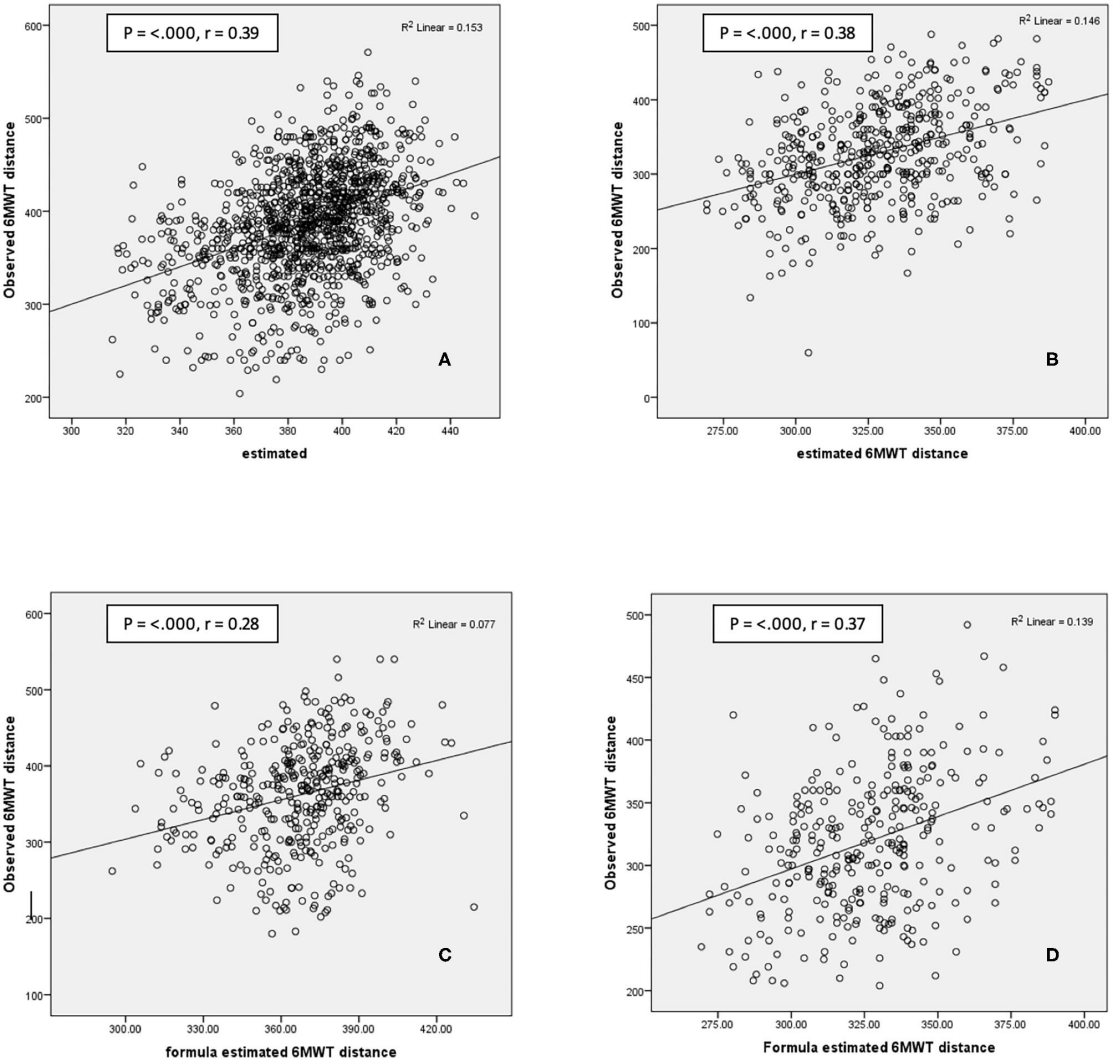
Scatterplot of predicted value with actual 6-MWT. Internal validation: **(A)** CABG surgery group 2014–2016 population; **(B)** valve surgery group 2014–2016 population. External validation: **(C)**. CABG surgery group 2018 population. **(D)** Valve surgery group 2018 population. *p*, Pearson correlation significant; *r* = Pearson coefficient; 6-MWT, 6-min walk test; CABG, coronary artery bypass graft.

**Figure 3:**
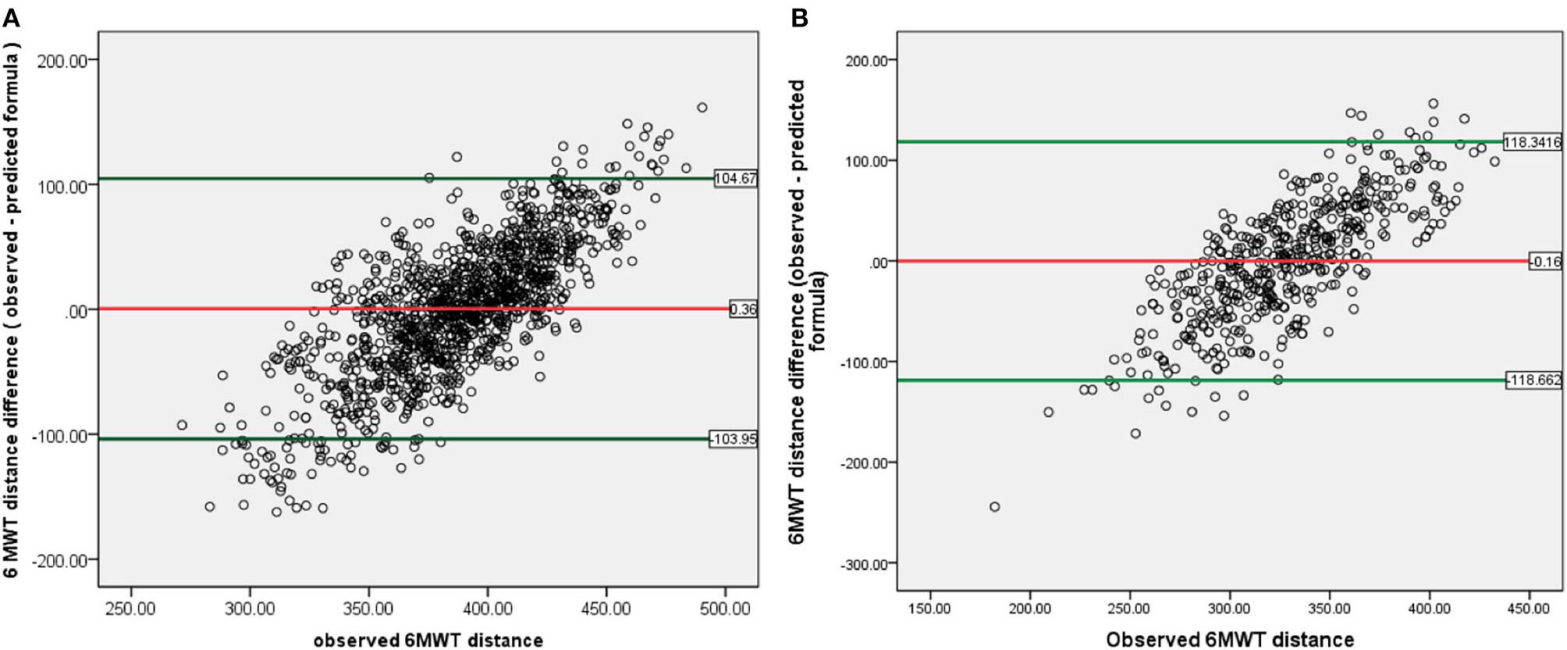
Bland–Altman plot of 6-MWT distance difference between observed and predicted formula, against actual observed 6-MWT distance for 2014–2016 population: **(A)** CABG group. **(B)** Valve surgery group. ^*^Red line indicates mean difference between 6-MWT distance predicted and observed and showed values close to 0 for both groups. Green line indicates upper and lower limit agreement of difference in the population. 6-MWT, 6-min walk test; CABG, coronary artery bypass graft.

The correlation scatter plot for each population and each group are presented in Figure 2; Pearson's product moment correlation shows significant result (*p* < 0.05) with coefficient correlation approximately 30–40%. The Bland–Altmann plot (Figure 3) also shows that the average differences between predicted and observed 6-MWD is close to 0 for the CABG group and the valve surgery group (0.36 and −0.16 m, respectively), which indicates no significant difference between the predicted 6-MWD from the equations and the observed 6-MWTdistance.

The upper and lower limit variance agreement of the difference in this study was between −103.95 and +104.67 m for the CABG group and between −118.86 and +118.34 m for the valve surgery group. There was a tendency of proportional bias with bigger difference variance at lower and higher observed 6-MWD.

## Discussion

This study was conducted in an institution where the number of cardiac surgeries was the largest in the country. In this institution, the 6-MWT was used as the main functional capacity test before commencing or after finishing the early phase II of the CR program, with exceptions if the medical assessment indicated that the patients could safely perform maximum exercise stress test. The 6-MWT is selected because it is a safe, easy to perform, suitable, and reliable test for the patients after cardiac surgery or heart failure or patients who are unable to perform maximum treadmill or ergocycle test (6–8). Additionally, the 6-MWT also has a good correlation with measured oxygen uptake (VO_2_) (2, 9), so that it could be used to obtain a predictive value when cardiopulmonary exercise testing is unavailable. Besides those reasons, the patients in the institution register early to the CR program after the surgery (within the first week after hospital discharge) when a maximum exercise test is more challenging to perform.

This study has a mean 6-MWD that was 57.4 ± 12.8% of the predicted distance of the healthy population reference (5), which indicates a reduced functional capacity after cardiac surgery compared with the normal population. But when these values were compared to similar patients, it would be challenging to grade their functional capacity as there are still no available reference. From our knowledge, there are no published studies regarding the minimum clinically important difference (MCID) in 6-MWD after cardiac surgery. However, as these results were compared to a study by Spertus et al. (10) about 6-MWD MCID for chronic heart failure patients for moderate decline of global rating of change scale (mean 6-MWD of 90 m), majority (78% for the CABG group, 82% for the valve surgery group) of the 6-MWD differences between the predicted and observed distances in our population are still below the MCID limit mentioned.

Reduced functional capacity, as observed in this study, is a common condition in patients after cardiac surgery especially in older age and in women when measured using 6-MWT or direct gas analysis (11). Presumably, it might be caused by the summative effect of existing chronic diseases, the impacts of surgical procedure (chest and leg discomfort due to the surgical wound), long-term coronary artery disease (systolic or diastolic dysfunction), pulmonary hemodynamic changes due to valvular heart diseases, previous speed of habitual walking or cultural aspects related to lifestyle, mood, attitude, and motivation of the patients or technicians (2, 12). However, it is evident that there will be a significant increase of functional capacity after the comprehensive CR program (13). This functional capacity reduction cannot be interpreted whether they are higher or lower for patients after cardiac surgery, since there is no available reference. Ultimately, the equations from this study can be used to grade a patient's functional capacity after CABG or valve surgery.

The baseline characteristic of the patients in the CABG group and the valve surgery group were different (Table 1). In the CABG group, the patients were significantly older, and they had more atherosclerosis risk factors such as diabetes mellitus, being an active smoker, dyslipidemia, higher systolic blood pressure, and body weight. Meanwhile, in the valve surgery group, the mean ejection fraction is higher than in the patients in the CABG group, and they had more incidences of AF. The 6-MWD soon after surgery was also longer in the valve surgery group than in the CABG group. This condition is caused by the nature of the different processes between coronary artery disease and valvular disease.

The univariable analysis revealed that female gender, older age, and lower body height, together with AF, were related to shorter 6-MWD early after CABG and heart valve surgery. Diabetes also had a negative effect on 6-MWD soon after CABG but not for the valve surgery group.

The multivariable linear regression analysis revealed that in patients of the CABG group, the determinants of the prediction equation were gender, age, body height, diabetes, and AF. Meanwhile, for the valve surgery group, the determinants were gender, age, and AF. Our prediction models explained only approximately 15% (R^2^) of the observed variation of 6-MWD after surgery, which is moderately satisfactory.

The strongest predictor of 6-MWD in this study was gender, followed by heart rhythm (AF or sinus). The influence of gender group on the 6-MWD in our study is consistent with previous studies in the healthy population (12, 13). However, our finding differs from a study of Oliveira et al. where gender was not the determinant variable of the predicted 6-MWD after cardiac surgery (14). This difference might be caused by the difference in the population characteristic and ethnicity, where in the previous study mentioned the population comprised non-Asian patients.

Several studies revealed some determinants probably influenced functional capacity early after surgery such as CPB time, type of surgeries, previous functional capacity, body mass index, age, gender, or co-morbidity (11, 14, 15).

It is important to have a 6-MWD reference from various groups of patients to compare and to consider whether a value resulting from a measurement is within normal range or subnormal. This study successfully generates two reference equations for 6-MWD for patients after cardiac surgery (CABG or valve surgery) which can be used for a similar population group.

The limitation of this study is that the data only came from a single center, so that additional data from other centers in the country must be collected to establish a national reference, including the 6-MWD reference for healthy persons. There were no data regarding the patient's functional capacity prior to the surgeries so that their functional capacity improvements as the direct effect of surgeries could not be explored. Another limitation is that the predicted functional capacity measurement is very much affected by individual motivation and other psychological factors, which were beyond the scope of the current study. However, from the authors' knowledge, this is the first study about reference equation prediction models of patients after cardiac surgery in an Asian population with satisfactory internal and external validation. Further studies could then revolve among other determinants which were not discussed yet in this study, in which the current discovered formula could be used as a baseline and thinking foundation in more advanced future studies.

## Conclusion

In conclusion, several determinants for the 6-MWD were identified, and two reference equations for predicting 6-MWD in patients after CABG and valve surgery were generated. Regarding patient characteristics, CABG is performed on older subjects, males, and more prevalent on diabetes; on the other hand, the prevalence of AF seems to be significantly higher in the valve surgery group. Although different variables are seen to influence 6-MWT in these two groups, the male gender seems to be the strongest predictor in both groups, while older age and AF were negative predictors. Additional exclusive predictors were seen in the CABG group; these include higher body height as a mild positive predictor and presence of diabetes as a negative predictor. It is expected that the equations generated in this study would assist clinicians in predicting the level of functional capacity of patients immediately after cardiac surgery.

## Data Availability Statement

The datasets generated for this article are not readily available because the data is a part of Indonesian National Cardiovascular Center registry of Cardiac Rehabilitation, data would be provided in the presence of acceptable purposes, such as multicenter study. Requests to access the datasets should be directed to basuni_radi@hotmail.com.

## Ethics Statement

The studies involving human participants were reviewed and approved by The Committee on Institutional Review Board/Health Research Ethics of National Cardiac Center Harapan Kita Hospital with the following registration number: LB.02.01/VII/536/KEP 027/2021 on Cardiac Rehabilitation Patient Registry. The patients/participants provided their written informed consent to participate in this study.

## Author Contributions

BR contributed in providing research ideas and leading the research team. The full article writing and study operations were completed by AA, BD, RI, KT, and AS. Statistical consultations were provided by BS. All authors have equal and significant contributions toward the final accomplishments of this study.

## Conflict of Interest

The authors declare that the research was conducted in the absence of any commercial or financial relationships that could be construed as a potential conflict of interest.

## Publisher's Note

All claims expressed in this article are solely those of the authors and do not necessarily represent those of their affiliated organizations, or those of the publisher, the editors and the reviewers. Any product that may be evaluated in this article, or claim that may be made by its manufacturer, is not guaranteed or endorsed by the publisher.
