# Supplementary material for: Determinants and Prediction Equations of Six-Minute Walk Test Distance Immediately After Cardiac Surgery
Source: Front Cardiovasc Med. 2021 Aug 19;8:685673. doi: 10.3389/fcvm.2021.685673 (PMC8416754; doi:10.3389/fcvm.2021.685673)
Supplement: Supplementary file 1 [file Table_1.DOCX]

Supplementary Material

# Supplementary Table

Supplementary Table 1. Univariate analysis of 6-MWT distance after cardiac surgery

| Variables |  | 6 MWT distance after cardiac surgery | | | | | |  | |
| --- | --- | --- | --- | --- | --- | --- | --- | --- | --- |
|  | 6 MWT distance after CABG | | | | 6 MWT distance after valve surgery | | | | |
|  | linear regression | | Pearson correlation | | linear regression | | Pearson correlation | | |
|  | Y = b + ax | | p | R | Y = b + ax | | p | | R |
| Male (1) | 340 + 48x | | *0.000 | 0.217 | 310 + 33x | *0.000 | | 0.244 | |
| Diabetes (1) | 391– 20x | | *0.000 | -0.139 | 328 – 1.8x | *0.486 | | -0.002 | |
| Dyslipidemia (1) | 383 – 1.1x | | 0.346 | -0.001 | 328 – 9.1x | 0.127 | | -0.051 | |
| Smoker (1) | 376 +11x | | *0.007 | 0.064 | 325 + 14x | *0.032 | | 0.083 | |
| ECG with AF (1) | 383 – 19x | | *0.018 | -0.054 | 335 – 15x | *0.008 | | -0.112 | |
| Age | 514 – 2.2x | | *0.000 | -0.203 | 380 – 1.2x | *0.000 | | -0.236 | |
| Systolic BP | 392 – 0.08x | | *0.027 | -0.050 | 297 + 0.28x | *0.013 | | 0.101 | |
| Diastolic BP | 336 + 0.7x | | *0.002 | 0.074 | 291 + 0.55x | *0.009 | | 0.106 | |
| Heart rate | 378 + 0.05x | | 0.182 | 0.023 | 323 + 0.05x | 0.389 | | 0.013 | |
| % LVEF | 373 + 0.14x | | 0.396 | 0.007 | 330 – 0.05x | 0.402 | | -0.011 | |
| Body height | 22 + 2.2x | | *0.000 | 0.180 | 27 + 1.8x | *0.000 | | 0.243 | |
| Body weight | 336 + 0.7x | | *0.000 | 0.108 | 322 + 0.1x | 0.105 | | 0.057 | |

CABG, coronary artery bypass graft; LVEF, left ventricle ejection fraction; ECG, electrocardiogram; AF, atrial fibrillation; For nominal data : male gender (1), diabetes (1), dyslipidemia (1), smoker (1). *Significant univariate correlation at p value < 0.05
